# Perinatal psychiatric episodes: a population-based study on treatment incidence and prevalence

**DOI:** 10.1038/tp.2016.190

**Published:** 2016-10-18

**Authors:** T Munk-Olsen, M L Maegbaek, B M Johannsen, X Liu, L M Howard, A di Florio, V Bergink, S Meltzer-Brody

**Affiliations:** 1 Department of Economics and Business Economics, National Center for Register-based Research, Aarhus University, Aarhus, Denmark; 2 CIRRAU-Centre for Integrated Register-based Research, Aarhus University, Aarhus, Denmark; 3 Health Service and Population Research Department, Institute of Psychiatry, King's College London, London, UK; 4 Division of Psychological Medicine and Clinical Neurosciences, Cardiff University, Cardiff, UK; 5 Department of Psychiatry, University of North Carolina at Chapel Hill, Chapel Hill, NC, USA; 6 Department of Psychiatry, Erasmus Medical Centre, Rotterdam, The Netherlands; 7 UNC Center for Women's Mood Disorder, Department of Psychiatry, The University of North Carolina at Chapel Hill, Chapel Hill, NC, USA

## Abstract

Perinatal psychiatric episodes comprise various disorders and symptom severity, which are diagnosed and treated in multiple treatment settings. To date, no studies have quantified the incidence and prevalence of perinatal psychiatric episodes treated in primary and secondary care, which we aimed to do in the present study. We designed a descriptive prospective study and included information from Danish population registers to study first-time ever and recurrent psychiatric episodes during the perinatal period, including treatment at psychiatric facilities and general practitioners (GPs). This was done for all women who had records of one or more singleton births from 1998 until 2012. In total, we had information on 822 439 children born to 491 242 unique mothers. Results showed first-time psychiatric episodes treated at inpatient facilities were rare during pregnancy, but increased significantly shortly following childbirth (0.02 vs 0.25 per 1000 births). In comparison, first-time psychiatric episodes treated at outpatient facilities were more common, and showed little variation across pregnancy and postpartum. For every single birth resulting in postpartum episodes treated at inpatient psychiatric facilities, 2.5 births were followed by an episode treated at outpatient psychiatric facility and 12 births by GP-provided pharmacological treatment. We interpret our results the following way: treated severe and moderate psychiatric disorders have different risk patterns in relation to pregnancy and childbirth, which suggests differences in the underlying etiology. We further speculate varying treatment incidence and prevalence in pregnancy vs postpartum may indicate that the current Diagnostic and Statistical Manual of Mental Disorders-5 peripartum specifier not adequately describes at-risk periods across moderate and severe perinatal psychiatric episodes.

## Introduction

Psychiatric episodes during pregnancy and following childbirth are complex, as the well-being of both the mother and infant must be considered in all treatment decisions. There is general consensus that childbirth triggers new postpartum episodes that can be on a spectrum of mild-to-severe presentation. The severe episodes affect 1–2 per 1000 new mothers,^1, 2, 3^ and have been described and quantified in a range of diverse studies using different data sources and study designs, for example, refs 1, 2, 4, 5, 6. Episodes requiring psychiatric hospitalization and specialist treatment in secondary and tertiary health-care systems are thought to be on the severe end of the spectrum. They are often characterized by severe depression with suicidal ideation,^7^ or as postpartum psychosis that has been closely associated with bipolar affective disorders,^8^ although it does not have a specific diagnoses.^9, 10^ Adding to this, a proportion of more moderate postpartum psychiatric episodes will be treated in primary care with prescription drugs. Although the threshold for using pharmacological therapy is different in the perinatal period due to concerns about possible risks to the fetus and breastfeeding infant, antidepressants are used as one treatment option for perinatal depression, particularly non-psychotic moderate-to-severe depression.^11^ Antidepressant use in the perinatal period has increased over time,^12, 13^ as it has for depression outside the perinatal period. Furthermore, the prevalence of antidepressant use in pregnancy varies significantly between countries and across studies: 3% in Denmark^14^ and 13% in the United States,^15^ overall challenging direct comparisons across time periods and between studies. Adding to this, evidence suggests that some perinatal psychiatric episodes will be unnoticed and consequently untreated,^16, 17, 18^ with one recent study highlighting that pharmacological treatment is less likely to be prescribed in perinatal illnesses compared with other time points.^19^

Together, postpartum psychiatric episodes comprise a range of different disorders and symptom severity,^11, 20^ which subsequently are diagnosed and treated in a range of treatment settings. This poses a significant challenge to the investigation of incidence of postpartum psychiatric episodes, as the data from various settings are needed. An additional challenge is that the most available data in the field does not differentiate between first-time episodes vs recurrent episodes, which in epidemiological terms can be described as mixing incident and prevalent case groups. Finally, past diagnostic specifiers in the Diagnostic and Statistical Manual of Mental Disorders (DSM) and International Classification of Diseases (ICD) diagnostic systems vary in terms of defining postpartum onset ranging from 4 to 6 weeks postpartum,^21, 22^ with the recent shift from DSM-4 to DSM-5 to also include psychiatric episodes in pregnancy as well as postpartum episodes.^21, 23^

To date, no studies have quantified the proportion of psychiatric episodes occurring during pregnancy and postpartum treated in a range of health-care settings including inpatient psychiatry, outpatient psychiatry and outpatient primary care. Similarly, no studies have jointly compared number of mothers with first-time episodes to recurrent episodes to make a comprehensive overview. Therefore, in the present study, we aimed to conduct a descriptive summary of first-time and recurrent psychiatric episodes across diagnostic categories in pregnancy and the postpartum period using various Danish population registers. We examined psychiatric disorders around childbirth within Danish health-care systems, including psychiatric inpatient and outpatient health-care treatment facilities, and prescriptions for psychotropic medication provided by family doctors in primary care as a proxy measure for moderate perinatal psychiatric episodes.

## Materials and methods

### Study design

The present study was designed as a descriptive prospective study based on the information from different Danish population registers. In designing this study, we aimed to present an overview of perinatal (pregnancy and postpartum) psychiatric episodes measured as incidence and prevalence of the combined records of women treated in various primary and secondary/tertiary health-care settings measured as either first-time or recurrent contacts. This was done for women born 1955 or later, who had records of one or more singleton births from 1 January 1998 until 31 December 2012.

### Data sources

Three different population registers provided information for the present study, and the information was linked through the CPR number, which is a personal identification number assigned to all citizens in the country.^24^ Women were identified through the Civil Registration System, which was initiated in 1968 and holds updated information on vital status (dates of birth, migration and death) and links to legal family members.^24^ Further data sources were: The Psychiatric Central Register, with available information on inpatient treatment since 1969 and outpatient treatment since 1995;^25^ and The prescription registry with available information on general practitioner (GP) provided prescriptions since 1995.^26^

### Identification of women, births and perinatal psychiatric episodes

We identified 1 908 494 women born in Denmark from 1 January 1955 and onwards, and considered only those with one or more singleton childbirths later than 1 January 1998. In total, we had 822 439 children born to 491 242 unique mothers. When looking at first-time and recurrent records, we considered each month, beginning at 9 months before birth until 12 months after birth with 1 month being defined as 30 days. By restricting to childbirths after 1 January 1998, we ensured a washout period of at least 2 years and 3 months where women were medication free, which was done to ensure correct classification of first-time prescriptions. Among the identified women, we examined each individual pregnancy and childbirth, and for women with multiple children, we obtained information regarding mental health for each of the recorded individual childbirths. For all births, we identified a pregnancy and postpartum period, defined as the period of time from 9 months before to date of birth (pregnancy) and from date of birth through 12 months after birth (postpartum).

In Denmark, all diagnoses are recorded using the ICD-10 classification system since 1994, and before 1994, the ICD-8 classification system was used.^22, 27^ Perinatal psychiatric episodes were defined in one of the following ways: (1) any type of recorded and/or treated psychiatric diagnoses at psychiatric treatment facilities (both inpatient and outpatient settings) or (2) were prescribed either antidepressants or antipsychotics. More precisely, the treated psychiatric episodes were defined as either any type of F chapter ICD-10 code: mental and behavioral disorders at inpatient or outpatient treatment psychiatric treatment sites in Denmark. Similarly, we used equivalent ICD-8 codes: 290–315 before 1994, to identify whether ICD-10 contacts were incident or prevalent. Also, we identified psychiatric episodes treated with antidepressants or antipsychotics, using the following Anatomical Therapeutical Chemical classification codes: N03-N07, which covered all prescriptions from GPs treating women in primary care during the defined pregnancy and postpartum period. Note that prescription data were used as a diagnostic proxy measure for moderate perinatal psychiatric episodes identified and treated in primary care settings by GPs.

For our study, we defined three different pregnancy and postpartum psychiatric episode groups ranging in disease severity, which were identified and treated at three different treatment settings:

1. Severe perinatal psychiatric episodes. Treatment setting: psychiatric inpatient treatment facility (secondary and tertiary care). Definition: all recorded F-diagnoses (ICD-10: mental and behavioral disorders).
2. Moderate perinatal psychiatric episodes. Treatment setting: psychiatric outpatient treatment facility (secondary care). Definition: all recorded F-diagnoses (ICD-10: mental and behavioral disorders).
3. Moderate perinatal psychiatric episodes. Treatment setting: Medical doctor/GP clinics (primary care). Definition: pharmacological treatment/prescriptions: N03-N07 (Anatomical Therapeutical Chemical codes).

The above-mentioned categorizations were made to capture perinatal psychiatric episodes, which range in severity and subsequently will be treated at different treatment sites. The episodes were therefore named moderate and severe for clarification, with the distinction being based on the information regarding treatment sites.

### Statistical analyses

The outcome measures for the present study were calculation of incidence and prevalence of perinatal psychiatric episodes in the three different treatment settings described above. This was done to provide information about how many new cases of psychiatric episodes including all recorded psychiatric diagnoses were recorded around pregnancy and postpartum (incidence), compared with measuring how widespread pregnancy and postpartum psychiatric episodes are (prevalence). First-time cases (incidence) were counted as the first-ever contact to the specific treatment setting with the specific treatment or diagnoses within the time period from 9 months before through 12 months after birth. The incidence was then calculated for each birth as the number of incident cases for each month divided by the total number of births. Note that our incidence definition meant that we identified first-ever episodes, and that the women also had no records of treatment before conception (outside the perinatal period). To ensure that our calculation of first-time treatment was indeed incident treatment, we introduced a washout period excluding all women with records of treated psychiatric episodes at the different treatment settings before 1 January 1998. As the individual registers/data sources were established at different time points, this introduced varying washout period from 2 years (prescription data) up >25 years (psychiatric inpatient treatment data).

Recurrent (prevalent) contacts were defined as all registered contacts in any of the three treatment settings from 9 months before through 12 months after birth, where contacts were counted on a monthly basis. This meant that for each month, we considered whether there was any record of treatment, with recurrent cases only counted once for each month. Note that our prevalence definition meant that women could have had records of treated psychiatric episodes both in a previous perinatal period and/or outside a perinatal period. For each birth, the prevalence was calculated as the number of recurrent cases divided by the total number of births.

In addition, we calculated incidence proportion and prevalence of treated postpartum psychiatric episodes jointly for the first 3 months postpartum to present an overall estimate for the first-time period following childbirth, which is particularly associated with both first onset and recurrence of psychiatric episodes.^1^

As a means for further explanation, consider the following example: woman A experiences severe depression 3 months postpartum, which is the first-ever episode of any recorded psychiatric disorder. She is admitted and treated at a psychiatric inpatient facility. For the calculated incidences, she is then recorded with her first-time episode 3 months after childbirth and contributes to the calculated incidence for this particular month. If woman A is readmitted to psychiatric treatment 9 months postpartum, she will only be included in the calculation of incidence in the third month postpartum, whereas she will contribute information in the calculation of prevalence at both 3 and 9 months postpartum.

For information regarding the code used to generate the results, please contact the first author.

## Results

Based on the data covering 822 439 children born to 491 242 mothers, we studied the incidence and prevalence of postpartum psychiatric episodes in various treatment settings.

### Inpatient psychiatric treatment facilities

**Figure 1:**
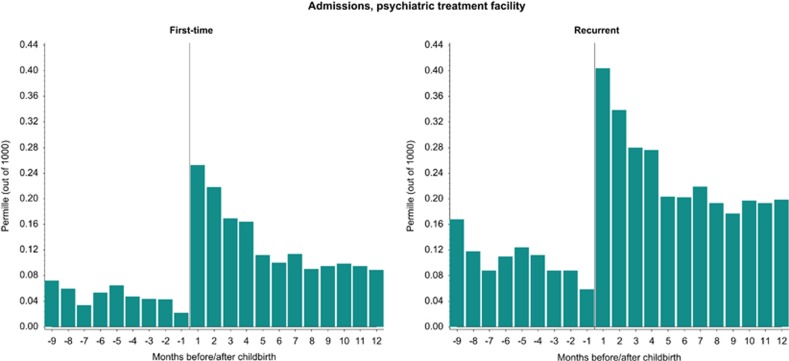
First-time and recurrent psychiatric episodes in the perinatal period treated at psychiatric inpatient facilities. Information on admissions to psychiatric treatment facilities came from the Psychiatric Central Register, with data available since 1969. *Y* axis: permille (out of 1000)=per 1000 births.

First-time and recurrent psychiatric episodes treated at psychiatric inpatient facilities were most common within the first month following childbirth, 0.25 and 0.40 per 1000 births (Figure 1). At any time during the observation period from 9 months before birth through 12 months following the lowest observed incidence and prevalence was the last month before childbirth, 0.02 and 0.06 per 1000 births (Figure 1).

### Outpatient psychiatric treatment facilities

**Figure 2:**
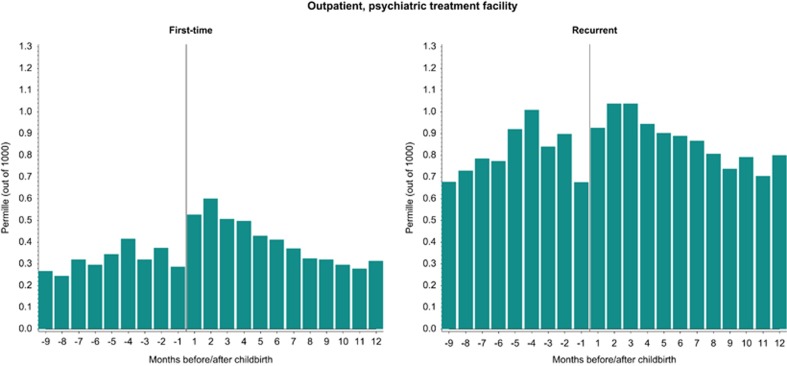
First-time and recurrent psychiatric episodes in the perinatal period treated at psychiatric outpatient facilities. Information on outpatient treatment at psychiatric treatment facilities came from the Psychiatric Central Register, with data available since 1995. *Y* axis: permille (out of 1000)=per 1000 births.

Between 31 and 60 days postpartum, the highest incidence of first-time psychiatric episodes treated at outpatient facilities was observed (0.60 per 1000 births, Figure 2). Both the incidence and prevalence of treated psychiatric episodes at outpatient clinics showed little variation when comparing the pregnancy period with the postpartum period (Figure 2).

### Primary care provided pharmacological treatment

**Figure 3:**
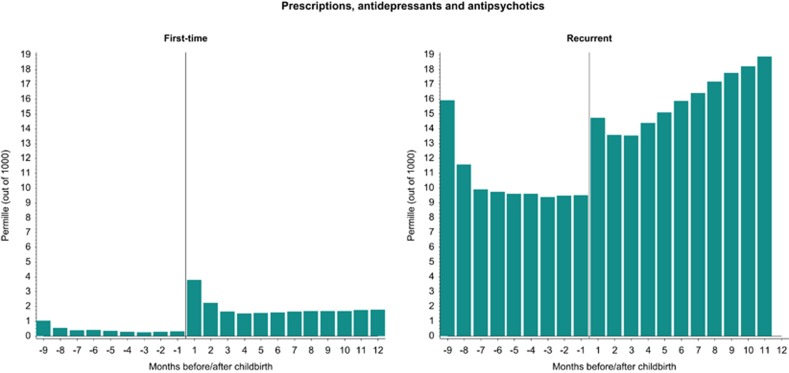
First-time and recurrent GP-provided prescriptions with antidepressants or antipsychotics in the perinatal period. Information on GP-provided prescriptions came from the Danish National Prescription Registry, with data available since 1995. *Y* axis: permille (out of 1000)=per 1000 births. GP, general practitioner.

First-time prescriptions with antidepressants and antipsychotics were most common within the first month postpartum (3.81 per 1000 births; Figure 3). In comparison, there was also a significant increase in prevalent prescriptions postpartum compared with the pregnancy period (14.74 per 1000 births; Figure 3).

### Incidence proportion and prevalence of perinatal psychiatric episodes

Overall measures of incidence proportion and prevalence 0–3 months postpartum are shown in Table 1. The incidence proportion 0–3 months postpartum for psychiatric episodes treated at inpatient psychiatric facilities was 0.64 per 1000 births. In comparison, the incidence proportion was 7.72 per 1000 births for psychiatric episodes treated in primary care with antidepressants and antipsychotics. This could be interpreted the following way: for every single birth resulting in postpartum episodes treated at psychiatric inpatient treatment facilities, 12 births are followed by a psychiatric episode treated with GP prescribed antidepressants or antipsychotics (7.72/0.64=12.06). For every birth resulting in postpartum episodes treated at inpatient treatment facilities, 2.5 are followed by a psychiatric episode treated at outpatient psychiatric treatment facilities (1.63/0.64=2.55). Adding to this, for every postpartum psychiatric episode treated in outpatient treatment facilities 4.7 are treated with antidepressants (7.72/1.63=4.74).

## Discussion

### Childbirth as a trigger for psychiatric episodes

Our data confirmed that childbirth is a potent and highly significant trigger of severe psychiatric episodes. We identified a rapid increase of particularly inpatient-treated episodes shortly following childbirth, and the first postpartum months were associated with the highest risks of both first-time and recurrent episodes. More precisely, we found that in 0.64 per 1000 births, mothers experienced new psychiatric episodes necessitating treatment at inpatient psychiatric treatment facilities 0–3 months postpartum. This estimate is lower than previously reported incidences of postpartum psychosis/severe postpartum psychiatric episodes, 1 per 1000.^1, 2^ We speculate that the reasons for this difference reflect changes across calendar time, as the number of available inpatient beds has decreased during the past decades,^28^ and possibly a subsequent shift toward treatment in outpatient clinic settings.

Applying an observation period of 3 months postpartum, we found that for every 1000 births the following numbers of psychiatric first-time episodes were treated: 0.64 inpatient psychiatric treatment, 1.63 outpatient psychiatric treatment and 7.72 primary care-treated moderate episodes treated with prescriptions of antidepressants/antipsychotics. This demonstrates that the majority of postpartum episodes are treated in primary care settings, but these numbers also reflect a difference in severity of the disorders, with inpatient admissions representing the most severe end of the spectrum.

The newest version of the DSM classification system introduced a peripartum specifier for disease onset both during pregnancy and postpartum,^23^ which is different from the previous definition for a specifier solely related to the postpartum period.^21^ The register data used for the present study are administrative and measures contacts with the health-care system, but not the onset of specific illness. Consequently, some women may have had the onset within the time put out by the DSM specifier. However, when observing our findings, the peripartum specifier may be a useful definition for the patterns of treatment especially in outpatient clinics (Figure 2), as we here did not observe wide variations between pregnancy and postpartum for the more moderate episodes. In comparison, when we examined data covering more severe episodes that required inpatient psychiatric treatment, the variation between the pregnancy period and postpartum was vast with a very low incidence in pregnancy and a clear peak in incidence shortly following childbirth (Figure 1). This may suggest that a peripartum specifier as used in the DSM system is not the most suitable definition for disorders in the most severe end of the spectrum, and we further hypothesize that these episodes could have different underlying etiologies than the less severe episodes. However, for other purposes such as service planning a peripartum specifier could be beneficial, and hence it is important to consider how a specifier is useful and which time period the definition should include.

Childbirth triggers first-ever severe episodes that are treated at inpatient psychiatric facilities (Figure 1). This is a particularly concerning observation, as we at present are not able to predict which women will become ill postpartum.^29, 30^ However, the majority of the treated severe postpartum episodes are recurrent episodes (Figure 1), meaning that the majority of women with severe episodes following childbirth are patients with previous records of treated psychiatric disorders outside the postpartum period. For these female patients, treatment and prevention plans should be available and initiated, especially if the individual women have histories of bipolar disorders or postpartum psychosis.^31^ Such plans could include medication prophylaxis, birth plan, intervention strategies from earliest signs of prodromal symptoms, neonatal medical evaluation, breastfeeding preferences and strategies for adequate sleep, maintenance of stable circadian rhythm, stress reduction, and support of maternal–newborn bonding.^31^

### Treatment to pregnant and postpartum women

Pharmacological treatment to pregnant and postpartum women has received a lot of attention, with concerns about the health of baby vs consequences of untreated mental disorders in the mothers posing the biggest dilemma.^32, 33^ Prescription patterns in the present study showed lower rates of use of antidepressants and antipsychotics during pregnancy and an increase following childbirth, but mainly for first-time prescriptions. This could indicate that Danish GPs are reluctant to prescribe medication to pregnant women due to concerns about teratogenicity and may choose non-pharmacologic treatment, including psychological intervention.^12, 34^ It is also possible that any increase in recorded prescriptions postpartum may not solely reflect a worsening of depressive symptoms, but also serves as a marker of treatment being postponed to after childbirth. This explanation is based on the observation that the rate of psychiatric medication prescription around time of conception is similar to the levels postpartum, especially for the recurrent cases. If this explanation is correct, any interpretation of the onset and recurrence of depressive symptoms during pregnancy is challenged. Importantly, there is no evidence that pregnancy is directly protective for the new occurrence or recurrence of psychiatric episodes. In our data, the decrease in prescribing of psychiatric medication during pregnancy was not compensated by an increase in outpatient treatments, which could include both pharmacological and non-pharmacological treatment options. Consequently, these findings suggest that many women with psychiatric episodes remain untreated during pregnancy. This is highly worrying, as untreated depression or other psychiatric episodes is associated with increased suicide risks in new mothers.^19^

In conclusion, our findings support and expand upon the previous work documenting that the postpartum period is a high-risk period for psychiatric episodes. In particular, childbirth especially triggers first-time severe psychiatric episodes requiring inpatient treatment and these episodes may require a distinct classification within diagnostic systems. In contrast, there is limited evidence for an increase in moderate psychiatric episodes postpartum compared with the pregnancy period, and for this category, the DSM-5 specifier ‘with perinatal onset' might adequately cover the at-risk period. Childbirth also triggers recurrence of severe psychiatric episodes, for which intervention and treatment plans should be prepared. Overall, our results suggest that there may be differences in the underlying etiology behind postpartum psychiatric episodes as we observed differences in the observed treatment incidence and prevalence for moderate versus severe episodes, as well as differences in risks during pregnancy vs postpartum.

## Figures and Tables

**Table 1:** Incidence proportion and prevalence 0–3 months postpartum for psychiatric episodes treated across the Danish health-care system

*Treated postpartum psychiatric episodes*	*Incidence proportion 0–3 months postpartum (per 1000 births)*	*Prevalence 0–3 months postpartum (per 1000 births)*
Psychiatric inpatient treatment facility	0.64	0.94
Psychiatric outpatient treatment facility	1.63	2.83
GP-provided prescriptions (antidepressants/antipsychotics)	7.72	29.35

Abbreviation: GP, general practitioner. 0–3 months=0–90 days postpartum.

Incidence proportion 0–3 months postpartum: defined as first-ever contacts=no previous contacts both in pregnancy and before conception.

Prevalence 0–3 months postpartum: If a woman, for example, had records of two prescriptions within 0–90 days postpartum, she was calculated twice for this measure. Note women can have had a previous record of treated psychiatric episode both perinatally and/or outside the perinatal period.
